# Association of low‐density lipoprotein receptor‐related protein 1 rs11613352 and angiopoietin‐like 3 rs2131925 with hypertension in men—the Tampere adult population cardiovascular risk study

**DOI:** 10.1002/mgg3.450

**Published:** 2018-07-10

**Authors:** Tarja Kunnas, Tiina Solakivi, Seppo T. Nikkari

**Affiliations:** ^1^ Department of Medical Biochemistry Faculty of Medicine and Life Sciences Fimlab Laboratories University of Tampere Tampere Finland

**Keywords:** ANGPTL3, FADS1, hypertension, LRP1

## Abstract

**Background:**

We examined the association of three known genome‐wide association study loci for blood lipids that have lead traits for triglycerides with hypertension in the Tampere adult population cardiovascular risk study.

**Methods:**

A Finnish cohort of 190 men with diagnosed hypertension and 279 controls were analyzed. Samples were genotyped for low‐density lipoprotein receptor‐related protein 1 rs11613352 (C>T), angiopoietin‐like 3 rs2131925 (T>G), and fatty acid desaturase 1 rs174546 (C>T) polymorphisms using competitive allele‐specific polymerase chain reaction technique.

**Results:**

At the age of 50, subjects with low‐density lipoprotein receptor‐related protein 1 rs11613352 (C>T) minor genotype TT had significantly more hypertension than those with the C allele (OR 5.17, CI 2.03–12.74, *p* < 0.001). Subjects with angiopoietin‐like 3 rs2131925 (T>G) T allele had more hypertension than those with the minor genotype GG (OR 5.02, CI 1.40–17.98, *p* = 0.013). Fatty acid desaturase 1 rs174546 (C>T) did not associate with hypertension.

**Conclusion:**

Association of low‐density lipoprotein receptor‐related protein 1 rs11613352 and angiopoietin‐like 3 rs2131925 with hypertension might imply a direct effect at the artery wall.

## 1 INTRODUCTION

Plasma concentrations of lipoproteins are important risk factors for cardiovascular disease. On the basis of GWAS studies, there exist a large number of common genetic variants that affect plasma lipoproteins (Teslovich et al., 2010). Although LDL‐cholesterol has remained the main lipid focus, plasma triglyceride (TG) concentrations have reemerged as a clinically significant cardiovascular disease risk factor (Goldberg, Eckel, & McPherson, 2011). An important question about such biomarkers is whether the genes associated with them are directly reflective of the cardiovascular disease process. We chose to examine the relationship of three TG‐associated genetic variants with hypertension on the basis that they might also affect the artery wall directly.

Previously known variants in GWAS with TG‐associations include fatty acid desaturase 1 (*FADS1*) (OMIM: 606148; GenBank NT_033903.7) rs174546 (C>T) (3′ UTR variant) (Teslovich et al., 2010). *FADS1* encodes the enzyme delta‐5 desaturase that modifies fatty acids to their respective polyunsaturated metabolites. Genetic variation in *FADS1* rs174546 influences blood pressure in young children via arachidonic acid formation (Wolters et al., 2017). New TG‐associated gene arrivals include the intronic locus rs2131925 (T>G), representing angiopoietin‐like 3 (ANGPTL3) (OMIM: 604774; GenBank NT_032977.10), a lipoprotein lipase inhibitor (Teslovich et al., 2010). A potential role in hypertension for ANGPTL3 is possible, since its blood levels have been shown to be closely associated with arterial wall thickness (Hatsuda et al., 2007). In addition, a single‐nucleotide polymorphism in the intronic locus rs11613352 (C>T) on chromosome 12q13.3 was associated with TG plasma levels (Teslovich et al., 2010). The locus has putatively been considered to represent the LDL receptor‐related protein 1 (LRP1) (OMIM: 107770; GenBank NT_029419.13), a member of the LDL‐receptor family (Aledo, Padro, Mata, Alonso, & Badimon, 2015). LRP1 has been proposed to have a direct effect at the artery wall (Strickland, Au, Cunfer, & Muratoglu, 2014), which might imply an association with hypertension.

We therefore examined whether the known above GWAS loci for triglycerides had association with hypertension in the TAMRISK study population.

## 2 MATERIALS AND METHODS

### 2.1 Subjects

TAMRISK study data were collected from periodic health examinations (PHE) done for 50‐year‐old men and women living in Tampere, a city in southern Finland with 220 000 inhabitants (Maatta, Nikkari, & Kunnas, 2015). Periodic health examination (PHE) was done in 2003 and included an interview by a public health nurse, using a structured questionnaire about health and health‐related behavior. The body mass index (BMI) was calculated from recorded height (cm) and weight (kg). Blood pressure (BP) measurement (mm of mercury) was done using a calibrated mercury sphygmomanometer. Serum TC, TG, and HDL‐C (from which LDL‐C was calculated) (mmoles/liter) were measured after an overnight fast by standard techniques. Buccal swabs for DNA extraction, and a permissions form to use PHE information were collected by mail separately of the physical examination during years 2006–2010.

Cases were subjects who had hypertension at the age of 50 years, and for each case, at least one normotensive control subject with the same sex and similar smoking habit was chosen in order of admission from the PHE cohort (Maatta, Nikkari, Lahteela, Palmroos, & Kunnas, 2013). Hypertension had been diagnosed by a physician by normal healthcare procedures. Physicians diagnose hypertension when blood pressure readings are consistently 140/90 mmHg or above. All subjects were ethnic Finns.

Because previous studies have suggested sex‐specific heritability of lipid traits (Weiss, Pan, Abney, & Ober, 2006), this study focused on men. The present study population included 466–469 men who had successful genotyping for the present three polymorphisms (276–279 controls and 189–190 cases).

### 2.2 Ethical compliance

Informed consent was obtained from all participants. The Ethics Committees of the Tampere University Hospital and the City of Tampere approved the study.

### 2.3 Genotyping

DNA was extracted from buccal swabs using a commercial kit (Qiagen Inc., Valencia, CA, USA). Genotyping was performed using KASP (competitive allelic‐specific amplification) genotyping services at KBioscience Institute, UK. The call rate was 92%–93%. Details of this method can be obtained from https://www.lgcgroup.com/genotyping/.

### 2.4 Statistical analysis

Logistic regression was used to obtain odds ratio (OR) and 95% confidence interval (CI) for association analyses of LRP1 rs11613352, ANGPTL3 rs2131925 and FADS1 rs174546 genotypes with hypertension. Association analyses of the different genotypes with lipids were done after combining the men with hypertension and their controls. One‐way ANOVA and *t* test were applied for the comparison of genotype groups. *p* values <0.017 were considered significant. Analyses were carried out using SPSS 23.0 for Windows (SPSS Inc., Chicago, IL, USA).

## 3 RESULTS

Clinical characteristics of the subjects in the TAMRISK study have been previously described (Maatta et al., 2015). In this subpopulation, the studied single‐nucleotide polymorphisms (SNPs) were common, with minor allele frequencies (MAF) ranging 0.24–0.40. The genotype frequencies of LRP1 rs11613352 (C>T; 0.57, 0.37, 0.06; MAF = 0.24), ANGPTL3 rs2131925 (T>G; 0.53, 0.41, 0.06: MAF = 0.26), and FADS1 (C>T; 0.36, 0.48, 0.16; MAF = 0.40) were in Hardy–Weinberg equilibrium (*p* = 0.823, *p* = 0.324 and *p* = 0.959, respectively). We analyzed the subpopulation of hypertensive men and controls whose clinical characteristics are shown in Table 1. Subjects with hypertension had significantly higher BMI, TG, systolic and diastolic blood pressure, and lower HDL‐C than controls.

**Table 1:** Clinical characteristics of the male study population at the age of 50 years

	Controls (*n* = 279)	Hypertension (*n* = 190)	*p* value^a^
Body mass index kg/m^2^ (*SD*)	25.6 (3.2)	29.3 (5.0)	0.000
Cholesterol, mmol/L (*SD*)	5.39 (0.94)	5.30 (1.00)	0.337
HDL‐cholesterol, mmol/L (*SD*)	1.52 (0.36)	1.40 (0.36)	0.001
Triglycerides mmol/L (*SD*)	1.36 (0.86)	1.74 (1.19)	0.000
LDL‐cholesterol, mmol/L (*SD*)	3.27 (0.82)	3.15 (0.90)	0.134
Systolic blood pressure, mmHg (*SD*)	130.3 (14.7)	143.7 (17.0)	0.000
Diastolic blood pressure, mmHg (*SD*)	85.7 (9.3)	93.6 (9.4)	0.000

a *T*‐test.

**Figure 1:**
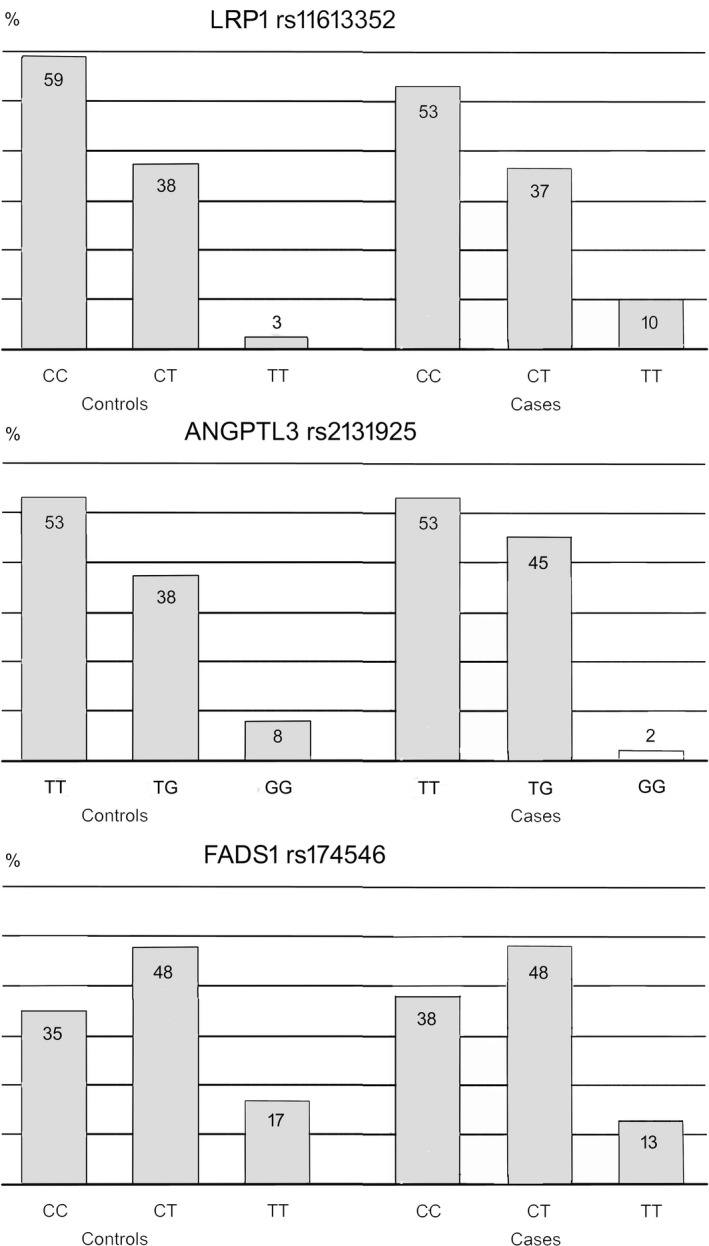
Bar graph showing distributions of *LRP1*,*ANGPTL3*, and *FADS1* polymorphisms in male cases with hypertension and their controls

Distributions of LRP1, ANGPTL3, and FADS1 polymorphisms in male cases with hypertension and their controls are shown in Figure 1. Association analyses of the genotype frequencies with hypertension for *LRP1* rs11613352 (C>T), *ANGPTL3* rs2131925 (T>G), and *FADS1* rs174546 (C>T) are given in Table 2. Subjects with *LRP1* rs11613352 (C>T) minor genotype TT had significantly more hypertension than those with the C allele. At the age of 50, even after adjusting for BMI, compared to C allele, genotype TT was associated with higher prevalence of hypertension (OR 5.17, CI 2.03–12.74, *p* < 0.001). Subjects with *ANGPTL3* rs2131925 (T>G) T allele had more hypertension than those with the minor genotype GG. When the T‐allele carriers were compared to the GG genotype, after adjusting for BMI, the OR for hypertension was 5.02 (CI 1.40–17.98, *p* = 0.013). *FADS1* rs174546 (C>T) did not associate with hypertension.

**Table 2:** Distributions of *LRP1*,* ANGPTL3*, and *FADS1* polymorphisms in male cases with hypertension and their controls

	Controls		Cases		Unadjusted			Adjusted by BMI		
	*n*	%	*n*	%	OR	95% CI	*p* value^a^	OR	95% CI	*p* value^a^
LRP1 rs11613352										
At the age of 50 years										
CC	164	59.4	101	53.2						
CT	104	37.7	70	36.8						
TT	8	2.9	19	10.0						
TT versus (CC+CT)					**3.72**	**1.59, 8.69**	**0.002**	**5.17**	**2.03, 12.74**	**<0.001**
ANGPTL3 rs2131925										
At the age of 50 years										
TT	149	53.4	101	53.4						
TG	107	38.4	85	45.0						
GG	23	8.2	3	1.6						
(TT+TG) versus GG					**5.57**	**1.65, 18.82**	**0.006**	**5.02**	**1.40, 17.98**	**0.013**
FADS1rs174546										
At the age of 50 years										
CC	97	35.1	73	38.4						
TC	131	47.5	92	48.4						
TT	48	17.4	25	13.2						
(CC+TC) versus TT					1.37	0.82, 2.31		1.28	0.73, 2.25	0.383

*Notes*

Frequency (*n*); CI: confidence interval; OR: odds ratio. *p* values <0.017 are in bold.

a Logistic regression.

Association analyses of the different genotypes with lipids were done after combining the men with hypertension and their controls (Table 3). No statistically significant associations with lipids for *LRP1* rs11613352, *ANGPTL3* rs2131925, or *FADS1* rs174546 were found in this male population.

**Table 3:** Lipid values (means ± *SD*) stratified according to *LRP1*,* ANGPTL3*, and *FADS1*

LRP1 rs11613352	CC	CT	TT	*p* value a CC versus CT versus TT	*p* value a CC versus (CT+TT)	*p* value a TT versus (CC+CT)
*n* at 50	265	174	27			
Cholesterol, mmol/L (*SD*)	5.27 (0.94)	5.43 (1.01)	5.40 (1.02)	0.202	0.075	0.733
HDL‐cholesterol, mmol/L (*SD*)	1.47 (0.39)	1.47 (0.34)	1.44 (0.29)	0.909	0.899	0.662
Triglycerides mmol/L (*SD*)	1.49 (0.98)	1.58 (1.15)	1.62 (1.10)	0.638	0.353	0.659
LDL‐cholesterol, mmol/L (*SD*)	3.16 (0.83)	3.27 (0.91)	3.23 (0.89)	0.448	0.210	0.854

ANGPTL3 rs2131925	TT	TG	GG	*p* value a TT versus TG versus GG	*p* value a (TG + GG) versus TT	*p* value a (TT + TG) versus GG
*n* at 50	250	192	26			
Cholesterol, mmol/L (*SD*)	5.38 (0.97)	5.28 (0.96)	5.22 (0.89)	0.440	0.216	0.510
HDL‐cholesterol, mmol/L (*SD*)	1.50 (0.36)	1.42 (0.35)	1.54 (0.42)	0.032	0.043	0.289
Triglycerides mmol/L (*SD*)	1.57 (1.10)	1.53 (0.99)	1.21 (0.83)	0.068	0.370	0.021
LDL‐cholesterol, mmol/L (*SD*)	3.22 (0.84)	3.19 (0.86)	3.14 (0.92)	0.876	0.660	0.708

FADS1 rs174546	CC	TC	TT	*p* value a CC versus TC versus TT	*p* value a (TC + TT) versus CC	*p* value a (CC + TC) versus TT
*n* at 50	170	223	73			
Cholesterol, mmol/L (*SD*)	5.29 (0.85)	5.409 (1.06)	5.224 (0.92)	0.263	0.444	0.264
HDL‐cholesterol, mmol/L (*SD*)	1.50 (0.37)	1.43 (0.35)	1.47 (0.38)	0.154	0.078	0.859
Triglycerides mmol/L (*SD*)	1.51 (1.08)	1.60 (1.07)	1.41 (0.88)	0.670	0.526	0.696
LDL‐cholesterol, mmol/L (*SD*)	3.15 (0.79)	3.27 (0.91)	3.15 (0.86)	0.357	0.300	0.547

*Notes*

Frequency (*n*); HDL: high‐density lipoprotein; LDL: low‐density lipoprotein; *SD*: standard deviation.

a One‐way ANOVA or *t* test for continuous variables.

## 4 DISCUSSION

Serum TG level is reemerging as an independent risk factor for cardiovascular disease (Johansen et al., 2011). Recent GWAS studies have identified a locus on chromosome 12q13.3 associated with plasma levels of TG and HDL‐C, with rs11613352 being the single‐nucleotide polymorphism (Teslovich et al., 2010). Several genes lie in the ±500 Kb region of the GWAS signal of rs116133523, and the functional variant associated with this polymorphism has putatively been considered to be LRP1, a member of the LDL‐receptor family (Aledo et al., 2015). We investigated the role of *LRP1* rs11613352 with hypertension in a Finnish population. Men with the rs11613352 (C>T) minor genotype TT had significantly more hypertension than those with the C allele. Aledo et al. found that the polymorphism rs11613352 may contribute to attenuate cardiovascular risk by modifying plasma lipid levels in FH patients, as the TT genotype displayed a profile of lower TG and higher HDL‐C (Aledo et al., 2015). The association for lipids was similar to the GWAS study (Teslovich et al., 2010). Although a lipid association was not seen in the present male population, we confirmed it for increased HDL‐C seen with the TT genotype in 50‐year‐old women in the TAMRISK population (*p* = 0.001 for comparison of genotypes, *n* = 300) (data not shown). However, there was no association for *LRP1* rs11613352 with hypertension in women, confirming previous studies suggesting sex‐specific heritability of lipid traits (Weiss et al., 2006). LRP1 may also act directly at the artery wall, since it has been shown that mice are prone to aneurysm formation when *LRP1* gene has been selectively deleted in vascular smooth muscle cells. A mechanism for this is that LRP1 may protect against elastin fiber fragmentation by reducing activity of various proteases in the artery wall (Strickland et al., 2014).

ANGPTL‐3 is a member of the angiopoietin‐like proteins, a family of secreted glycoproteins, expressed in the liver, which can inhibit lipoprotein lipase and endothelial lipase (Oldoni et al., 2016). Men with *ANGPTL3* rs2131925 (T>G) T allele had more hypertension than those with the minor genotype GG. Previously, the TT genotype has been shown to be associated with increased TG and LDL‐C (Teslovich et al., 2010). We found no statistically significant association with lipids in our male population. Because of its intronic nature, there is also strong likelihood that rs2131925 is simply acting as a marker for the true functional variant(s) (Oldoni et al., 2016).

*FADS1* rs174546 (C>T) did not associate with hypertension or lipids. FADS1 is involved in endogenous synthesis of unsaturated fatty acids that are known to modulate the metabolism of lipids and lipoproteins and therefore also to be involved in cardiovascular diseases (Das, 2008). Although an association with lipids was reported by GWAS studies (Teslovich et al., 2010), no association with lipids was observed also in another study for a population homozygous for the minor T allele of rs174546 that associates with lower FADS activity (Hellstrand et al., 2014). Likewise, rs174546 was only marginally associated with increased TG in a Chinese population (Zhang et al., 2011).

Strengths of the study include that all participating subjects were 50 years old when the PHE examination took place. Limitations of the study include a modest sample size and a restricted genetic pool.

In conclusion, the *LRP1* rs11613352 (C>T) minor genotype TT previously associated with a profile of lower TG and higher HDL‐C (Aledo et al., 2015) was associated with hypertension in men. For *ANGPTL3* rs2131925 (T>G), men with the T allele had more hypertension than those with the minor genotype GG. Previously, the TT genotype has been shown to be associated with increased TG and LDL‐C (Teslovich et al., 2010). Association of *LRP1* 1 rs11613352 and *ANGPTL3* rs2131925 with hypertension might imply a direct effect at the artery wall.

## CONFLICT OF INTEREST

None declared.
